# Polygenic and sex specific architecture for two maturation traits in farmed Atlantic salmon

**DOI:** 10.1186/s12864-019-5525-4

**Published:** 2019-02-15

**Authors:** Amin R. Mohamed, Klara L. Verbyla, Hawlader A. Al-Mamun, Sean McWilliam, Bradley Evans, Harry King, Peter Kube, James W. Kijas

**Affiliations:** 1 Commonwealth Scientific and Industrial Research Organisation Agriculture and Food, Queensland Bioscience Precinct, St Lucia Brisbane, 4067 Australia; 2 Commonwealth Scientific and Industrial Research Organisation Data 61, Canberra, Australian Capital Territory 2601 Australia; 3 Tassal Operations Pty Ltd, Hobart, Tasmania 7001 Australia; 4 Commonwealth Scientific and Industrial Research Organisation Agriculture and Food, Hobart, Tasmania 7004 Australia; 5 Zoology Department, Faculty of Science, Benha University, Benha, 13518 Egypt

**Keywords:** Atlantic salmon (*Salmo salar*), Sexual maturation, Genetic architecture, GWAS, SNP, *Picalm*

## Abstract

**Background:**

A key developmental transformation in the life of all vertebrates is the transition to sexual maturity, whereby individuals are capable of reproducing for the first time. In the farming of Atlantic salmon, early maturation prior to harvest size has serious negative production impacts.

**Results:**

We report genome wide association studies (GWAS) using fish measured for sexual maturation in freshwater or the marine environment. Genotypic data from a custom 50 K single nucleotide polymorphism (SNP) array was used to identify 13 significantly associated SNP for freshwater maturation with the most strongly associated on chromosomes 10 and 11. A higher number of associations (48) were detected for marine maturation, and the two peak loci were found to be the same for both traits. The number and broad distribution of GWAS hits confirmed a highly polygenetic nature, and GWAS performed separately within males and females revealed sex specific genetic behaviour for loci co-located with positional candidate genes phosphatidylinositol-binding clathrin assembly protein-like (*picalm)* and membrane-associated guanylate kinase, WW and PDZ domain-containing protein 2 (*magi2)*.

**Conclusions:**

The results extend earlier work and have implications for future applied breeding strategies to delay maturation in this important aquaculture species.

**Electronic supplementary material:**

The online version of this article (10.1186/s12864-019-5525-4) contains supplementary material, which is available to authorized users.

## Background

The development of sexual maturation in Atlantic salmon (*Salmo salar*) is a complex process informed by both genetic and environmental cues. It is highly variable, with extremes in age and size at maturation the result of adaptation to maximise fitness and reproductive success [1]. The trait reflects a trade-off, whereby older maturing animals have higher reproductive success but an elevated risk of mortality prior to reproduction [2]. Life-history strategies typically include one or more years of freshwater residence post-hatch followed by multiple years at sea, before the commencement of maturation and migration to the native river for spawning. A proportion of fish stay at sea only 1 year and mature early (grilse). Further, male juveniles (parr) can mature precociously in freshwater, bypassing the marine phase completely [3]. Factors influencing the timing of maturation, including temperature and photoperiod manipulation are described in detail elsewhere [4].

The practise of salmon farming promotes high growth and adiposity through increasing food availability in comparison to wild stocks. The consequence is an elevated proportion of farmed animals entering maturation younger and at weights below harvest size, generating substantial production inefficiency. This can be partly addressed through modified management practise and selective breeding for delayed maturation, however the positive correlation with growth makes uncoupling positive and negative production impacts challenging. Existing estimates indicate the age and size of maturation are moderately heritable, ranging from 0.15 to 0.48 [5]. Genome wide association studies seeking to identify genes regulating disease and production traits are now becoming routine for farmed Atlantic salmon following the development of SNP genotyping platforms [6–10]. The first GWAS to investigate age at sexual maturation used a SNP tool comprising 6.5 K loci [7]. The study reported separate associated genome regions controlling grilsing and late maturation, suggesting the traits may be under independent genetic control. More recently, sequence based GWAS has made significant progress in wild populations through the comparison of one and three sea winter maturation animals. These two studies identified a single locus in European Atlantic salmon associated with age at maturity [11, 12]. The causal gene is likely to be the vestigial-like family member 3 gene (*vgll3*), which has a role in both regulation of adiposity and age at menarche in humans [13].

The objective of this study was to characterise the distribution of SNP effects that control variation in the timing of maturation, and identify the genes and genomic regions likely to regulate two maturation traits in salmon farmed in Tasmania. A custom SNP50 genotyping array was used to perform GWAS for 2721 fish with maturation data collected in the marine environment, while a parallel GWAS used 1846 related fish scored for maturation in freshwater. The results represent a detailed view of the genomic regions that explain variation in both traits, and provide key information for the design of selective breeding approaches to manage a substantial production challenge for the salmon farming industry.

## Methods

### Animals and trait measurements

The GWAS population is from the Salmon Enterprises of Tasmania (SALTAS) selective breeding program which has been described previously [14–16]. A subset of individuals from the 2012, 2013 and 2014 year classes were measured for one of two traits. Maturation in the freshwater environment (FMAT) was collected for 1867 progeny at 22 months by visual assessment for the emergence of secondary sexual characteristics. This included development of a kype and altered coloration, meaning FMAT is a binary trait scored as either immature or mature. Progeny were from a total of 374 families. Sibs of this cohort were transferred to sea cages at approximately 13 months of age and grown for a further 9 months, before being assessed for maturation in the marine environment (MMAT) using the same method. A total of 2739 animals were scored for MMAT at 22 months of age, derived from 554 families.

### Genome wide association analysis

Fin clips were used for the extraction of DNA by a commercial provider (Center for Aquaculture Technologies, San Diego, California). Genotyping was performed using a custom 50 K Affymetrix SNP array by the same commercial provider, containing loci largely derived from an earlier custom 220 K SNP Affymetrix array developed by the Centre of Integrative Genetics (CIGENE) and AquaGen [11]. Sex for all progeny was assigned using intensity data from three DNA probe sets designed to detect the presence of exon 3 and exon 4 of sex-determining gene (*sdY*) [17]. Probes were included into the design of a custom 50 K array, and animals assigned as male where each *sdY* assay returned a positive signal, and as female where no intensity data was observed from any *sdY* assay. Raw genotype calls were quality controlled to remove i) SNP with sample call rate < 90%; ii) SNP with minor allele frequency < 1% and iii) samples with missing genotypes in excess of 5%. A comprehensive pedigree check was performed by comparing the coefficients of the additive relationship matrix and the genomic relationship matrix (G matrix) calculated via the first method described in [18]. A total of 39 fish were identified with multiple inconsistencies and removed. After quality control, 46,500 SNP remained for 4567 animals measured for either FMAT (1846) or MMAT (2721). Association analysis was carried out using mixed linear method analysis (MLMA) [19]. To reduce the rate of false positive associations, the approach includes construction of a genetic relationship matrix (GRM) to identify family based structure before reducing the contribution of closely related individuals during estimation of the test statistic. To avoid double fitting trait associated SNP in the model, the GRM was estimated with candidate markers excluded (MLMAe) using the MLMA-LOCO method as implemented in the Genome-wide Complex Trait Analysis (GCTA) [20]. This reduced the inflation of observed association signals compared with other methods such as linear regression (Additional file 1). Analysis for both traits (MMAT and FMAT) was performed using all animals after fitting sex as a covariate. Sex specific analyses were also performed. The data was divided into male (1884) and female (2820) subsets measured for FMAT (670 males, 1317 females) and MMAT (1214 males, 1503 females). Analyses were performed for each trait and sex subset. Permutation testing was used to define significance thresholds for the trait and sex specific analyses [21]. One thousand permutation tests were performed for each analysis by permuting the phenotypic values. Chromosome specific thresholds were calculated by taking the maximum test statistic after each permutation for each chromosome. The distribution of those maximum values after the 1000 permutations was then used to calculate the threshold. Thresholds with a significance level of 0.05 were used. The genome wide threshold was not used as it was driven by a higher number of associations on a select small number of chromosomes. Estimates of the proportion of genetic variance (%V_G_) explained by SNP with significant associations were established by fitting all significant SNP, the GRM used in the association analysis and a pedigree containing all relationships within the population in generalised linear mixed models in ASReml-R [22]. The proportion of the genetic variance was calculated as the squared effect of the SNP divided by the estimate of the total genetic variance. SNP were mapped to ICSASG_v2 (accession GCA_000233375.4 [23]) to facilitate plotting of loci in genomic order. PLINK v1.9 [24] was used to estimate linkage disequilibrium as *r*^2^ for SNP pairs on Ssa25. This was performed after setting the LD value threshold to 0.0 to allow the estimation of values across the full range from one to zero (−-ld-window-r2). To perform fine-mapping using dense SNP collections, haplotype phasing and imputation to sequence across chromosome Ssa11 was carried out using Eagle version 2.3.2 [25] and Minimac3 [26] respectively. A reference panel was used for imputation, containing 19 animals sequenced to 30 fold depth of coverage [17]. All reference panel fish are from the Saltas selective breeding program (year class range 2005–2013), meaning they are direct relatives of the GWAS population. Genome sequence from reference animals were used to identify 358,683 variants across Ssa11 as described previously [17]. The 50 K array used for GWAS contained 801 SNP on Ssa11 common to the reference panel SNP set which were used to impute full Ssa11 sequence for 4606 animals from the GWAS population. The potential effect on imputation when using only 19 animals in the reference panel prompted us to filter the imputed data based on the *R*^2^ value produced by Minimac3. SNP having *r*^2^ < 0.4 were removed, leaving 10,940 SNP. The dataset was further filtered to a final set of 458 common SNP spanning the 3 Mb critical interval (Mb 80–83) on Ssa11. Association analysis was carried out using linear regression as implemented in PLINK v1.9 [24].

### RNA-Seq analysis of salmon tissues

RNA-Seq datasets from eight Atlantic salmon tissues (brain, gut, liver, muscle, skin, spleen, ovary and testis) were downloaded from Sequence Read Archive (SRA) database (Additional file 2). Raw reads were mapped to ICSASG_v2 [23] using default parameters of BOWTIE [27] as implemented in TopHat2 version 2.1.1 [28]. Binary alignment map (BAM) files were converted into sequence alignment map (SAM) files using SAMtools version 1.4 [29]. Python package HTSeq version 0.7.2 [30] was applied to count unique reads mapped to exons. Raw counts were analyzed using the edgeR package [31] in the R statistical computing environment [32]. Count raw data for each of the eight tissue samples were normalized using the scaling normalization method implemented in edgeR to achieve cross-sample normalization. The trimmed mean of M-values (TMM algorithm) [31] was subsequently used to calculate the scaling factors according to the library size of each sample. Following edgeR normalization, the expression values FPKM (Fragments Per Kilobase of transcript per Million mapped reads) were log_2_ transformed. Transformed FPKM data for candidate genes were obtained and plotted as heat map using the R package pheatmap (https://cran.r-project.org/web/packages/pheatmap/index.html).

## Results

### Study population and two maturation traits

**Fig. 1:**
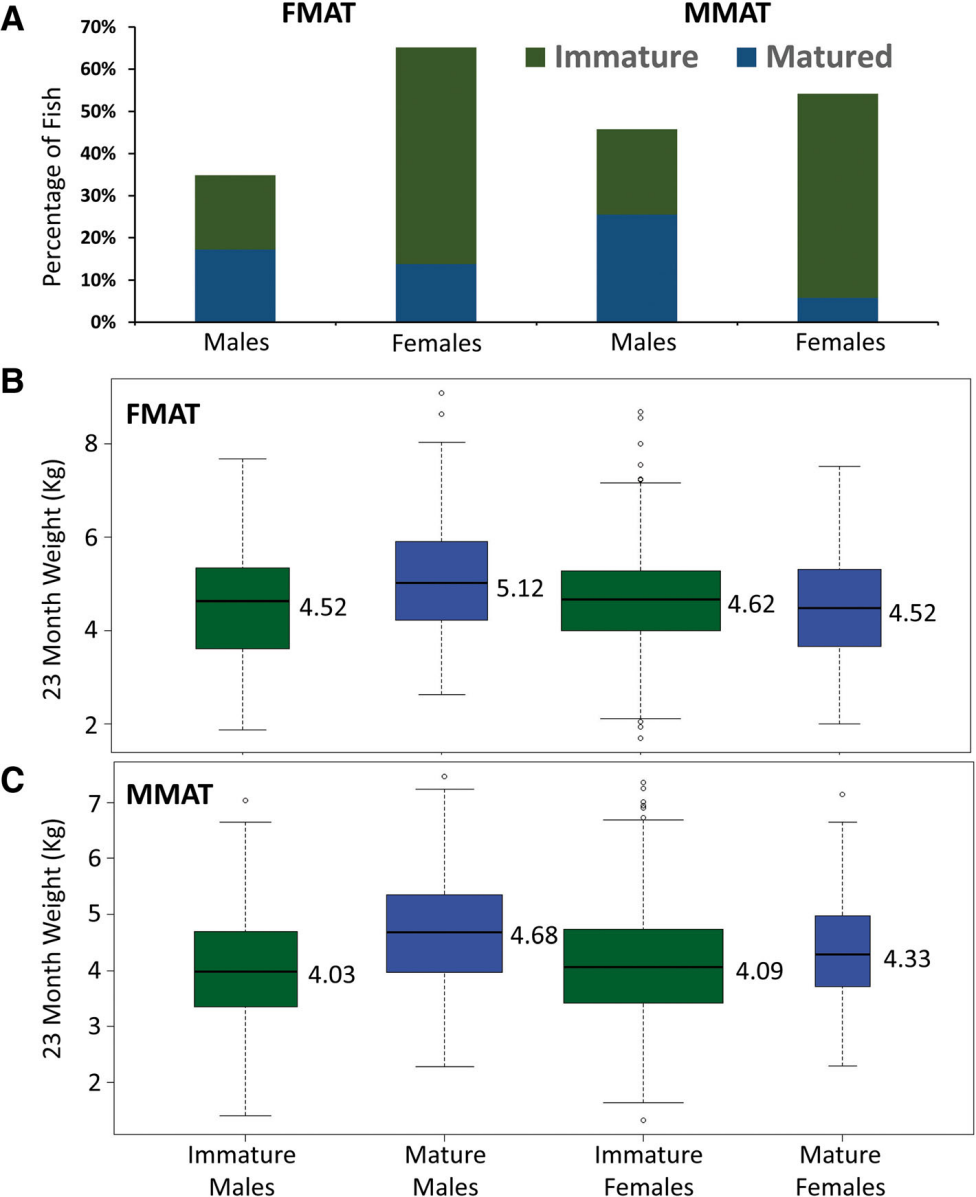
Relationship of sex and weight for two maturation traits. The observed proportions of mature and immature fish are shown for each sex and trait (**a**). The distribution of weight is shown separately as a function of both sex and maturation status for freshwater (**b**) and marine maturation (**c**)

The study population is from the SALTAS selective breeding program, which offers the opportunity to exploit large pedigrees and progeny trait data to perform GWAS. Maturation was assessed as two traits due to the life cycle of Atlantic salmon and the associated breeding program design (Additional file 3). Maturation in freshwater (FMAT) was assessed in 1846 progeny from 374 families at 22 months of age and scored as a binary trait. Matured individuals (*n* = 534 or 29%) were identified by visual assessment of secondary sexual characteristics such as the development of a kype and altered coloration. A significant sex bias was observed in early maturing animals, consistent with previous findings [11]. For example, around half of males had matured at 22 months of age compared with only 20% of females (Fig. 1, *χ*2 = 141, *p*-value = 1.0 × 10^− 32^). The weight of mature animals was no different among females. However, males maturing in freshwater were significantly heavier (Fig. 1; *p*-value = 1.9 × 10^− 9^). These biases were stronger for marine maturation (MMAT) which was measured in a separate group of 2721 animals from 554 families at 22 months of age, approximately 9 months after their transfer into sea cages. For example, only 10% of female fish presented as mature and they were significantly heavier than their immature female relatives (Fig. 1; p-value = 0.0086). In excess of half of the males had matured in the marine environment (55%) and they were also significantly heavier (Fig. 1; p-value = 8.7 × 10^− 25^). This is consistent with maturation in wild populations, where selection favours earlier maturation in males compared with females [11]. High accuracy family based heritability estimates were obtained. This revealed heritabilities at the lower end of the range amenable to GWAS (FMAT *h*^2^ = 0.15 ± 0.1; MMAT *h*^2^ = 0.20 ± 0.2). The correlation of family means was estimated to determine if the traits should be considered as biologically independent. This revealed the traits are only moderately correlated (*r*^2^ = 0.76), prompting their subsequent treatment in GWAS as independent.

### SNP association for freshwater maturation

**Fig. 2:**
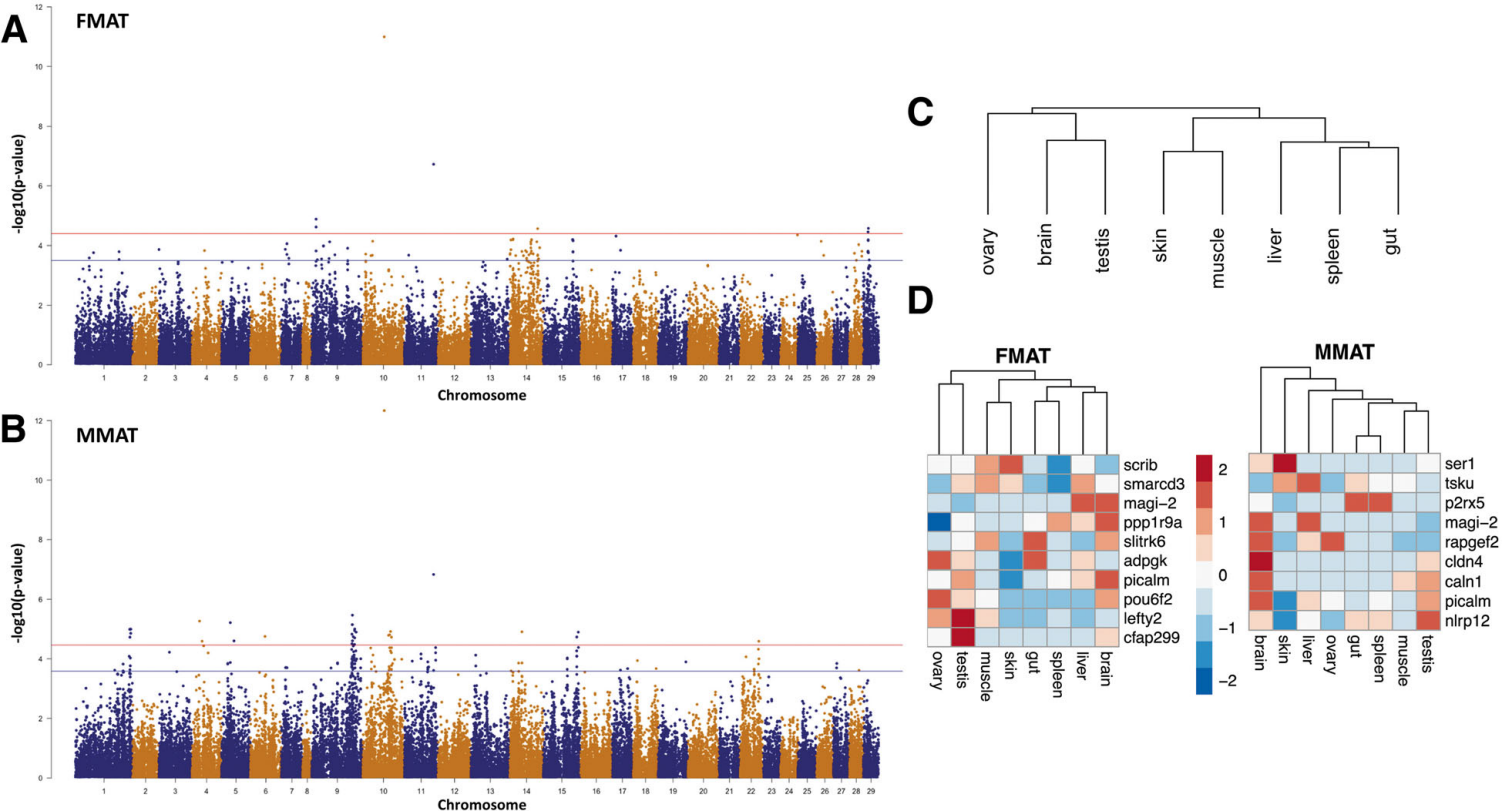
GWAS for two maturation traits in Atlantic salmon. SNP associations with freshwater (FMAT, **a**) and marine maturation (MMAT, **b**) are shown in genomic order for a karyotype consisting of 29 autosomes. The strength of association is given as the –Log10(*p*-value) and the horizontal lines represents the genome wide (red) or chromosome wide (blue line) significance thresholds. Expression levels from 45,531 genes was used to cluster a set of eight Atlantic salmon tissues. This was compared with heat maps of tissue-specific expression observed using positional candidate genes obtained from GWAS for FMAT (**d**) and MMAT (**e**). The values used are the log_2_ transformed fragment per kilobase million estimates (refer to the materials and methods)

To commence GWAS, genotyping was performed using a custom 50 K SNP array before quality filters were applied to define a total of 46,500 SNP. Analysis of both traits was performed using mixed linear model association incorporating a genetic relationship matrix. This is important for populations with family substructure, to reduce the over-estimation of significance and the incidence of false positive association. The strength of SNP association, estimated using 1846 genotyped animals scored for FMAT, is given in Fig. 2. A total of only 13 SNP were significantly associated, suggesting a polygenic trait. Significantly associated loci were distributed across the genome, with the ten most extreme loci on Ssa 9, 10, 11, 14, 17, 24 and 29 (Table 1). The highest ranked SNP was located at Mb position 60.2 on Ssa10 (*AX-87354755*,−Log *p*-value = 10.99), 169 Kb away from the *magi2* gene. It explains a moderate proportion of genetic variance (17.7%) with an effect size larger than other strongly associated loci (Table 1). The gene belongs to the guanylate kinase superfamily and exhibits a pattern of gonad expression that strongly suggests a role in ovarian differentiation [33]. The second most strongly associated SNP (Ssa11, *AX-96411005*, −Log *p*-value = 6.73) is located within the phosphatidylinositol-binding clathrin assembly protein-like gene *picalm*. It explains a lower proportion of genetic variation and has a smaller effect size (Table 1), however it has a known role in spermiation and is responsive to both estrogen and androgen when assayed in mouse seminiferous tubule culture. This suggests a role in mediating hormone regulation in reproductive tissues [34, 35]. While regulators of ovarian development (*magi2*) or steroid hormones (*picalm*) represent plausible positional candidates, there is currently little known about their role in fish. The identity of the top 10 ranked SNP for freshwater maturation, along with physically co-located genes, are given in Table 1 and Additional file 4.

**Table 1:** Top 10 most strongly associated loci for freshwater maturation (FMAT)

Ssa	SNP	Position	-LOG10(p-val)	Effect size	% V_G_	Gene
10	AX-87354755	60,175,081	10.99	−0.13	17.69	*magi2*
11	AX-96411005	81,521,399	6.73	−0.08	4.63	*picalm*
9	AX-87356700	11,857,001	4.88	0.07	6.92	*lefty2*
9	AX-87668311	11,873,490	4.62	0.07	0.02	*adpgk*
29	AX-87665166	13,717,091	4.57	0.09	6.47	*scrib*
14	AX-87530648	77,995,893	4.56	0.06	2.39	*ppp1r9a*
29	AX-87695835	12,876,814	4.45	0.07	2.01	*pou6f2*
24	AX-87594282	47,349,605	4.35	−0.12	7.98	*cfap299*
17	AX-96429168	10,920,789	4.31	0.08	5.32	*slitrk6*
29	AX-96491201	13,307,838	4.18	0.08	1.24	*smarcd3*

The chromosome number (Ssa), SNP identifier and base pair position of loci is given ranked using their strength of trait association given as –Log10(p-value). The effect size was derived from single SNP GWAS using the categoric assignment of mature or non-matured animals (0 or 1) at 22 months of age. The proportion of genetic variance (%V_G_) was estimated using a model fitting all significant SNP with both pedigree and SNP derived GRM. The complete of significant loci is provide in Additional file 4

### SNP association for marine maturation

The genomic distribution of associated SNP for marine maturation is also shown in Fig. 1 and listed in Table 2. A higher number of SNP exceeded the genome wide threshold for MMAT (48, Additional file 5) and the most significantly associated regions were located on chromosomes 6, 9, 10 and 11. The two highest ranked SNP for marine maturation are also the most significantly associated for freshwater maturation. The effect size for these SNP is similar for both traits, however the proportion of genetic variation explained was lower for MMAT compared with FMAT (Tables 1 and 2). Beyond these two loci on Ssa10 and Ssa11, there was no overlap of significantly associated SNP shared between the two traits. Other highly associated SNP for marine maturation include a broad association signal on Ssa9 (Mb 112.8–119.6, Table 2). The peak contains four of the ten most strongly associated loci genome wide, and each of the four SNP is located within a gene (*p2rx5*, *cld4*, *tsk* and *LOC106612817*). Of these, claudin-4 (*cld4*, *LOC106612824*) is a strong positional candidate as claudins are junction proteins and control electrolyte transportation across cell-cell junctions. Several claudins have roles in gonadal development such as claudin-3 which acts to control spermatocytes [36], while claudin-4 expression is responsive to estrogen receptor antagonists during development [37]. Human claudin-4 has been well studied due to over expression in ovarian cancer, and the gene’s normal function is important for ovarian follicular development [38].

**Table 2:** Top 10 most strongly associated loci for marine maturation (MMAT)

Ssa	SNP	Position	-LOG10(p-val)	Effect Size	% V_G_	Gene
10	AX-87354755	60,175,081	12.34	−0.11	2.52	*magi2*
11	AX-96411005	81,521,399	6.84	−0.07	2.64	*picalm*
9	AX-87290803	113,217,513	5.47	−0.06	6.36	*LOC106612812*
4	AX-87171059	21,354,256	5.27	0.12	13.93	*ctnna2*
5	AX-96304246	24,703,414	5.22	0.06	1.18	*rapgef2*
9	AX-87526812	112,774,492	5.15	−0.07	0.01	*cldn4*
9	AX-87822666	113,017,965	5.15	−0.07	LD	*caln1*
9	AX-96428686	119,632,095	5.00	−0.05	0.27	*tsku*
1	AX-87558959	154,144,608	5.00	0.12	3.11	*ser1*
1	AX-87477870	151,185,007	4.99	−0.06	0.97	*nlrp12*

The table has the same format as Table 1, and the complete list of all significant SNP for is provided in Additional file 5

The observation that the top ranked loci were shared between traits prompted us to attempt fine-mapping using additional loci. We focussed on the *picalm* gene on chromosome 11 as the associated SNP resides within the gene. The availability of whole genome sequence from 19 ancestors of the GWAS population [15] facilitated an imputation approach to saturate the region surrounding *picalm* with additional SNP. We imputed genotypes at 458 SNP in 4606 animals (1867 with MMAT and 2739 with FMAT trait records) across a 3 Mb region (Mb 80–83) spanning the Ssa11 peak SNP *AX-87621437* (Mb 81.52, Table 1). Repeating the GWAS with imputed SNP resolved the critical interval to a 7.9 Kb region containing 9 loci in complete linkage disequilibrium (Additional file 6). One of the 9 loci was *AX-87621437*, suggesting the genome wide peak SNP is located close to the causal variant.

### Sex specific genetic architecture

**Fig. 3:**
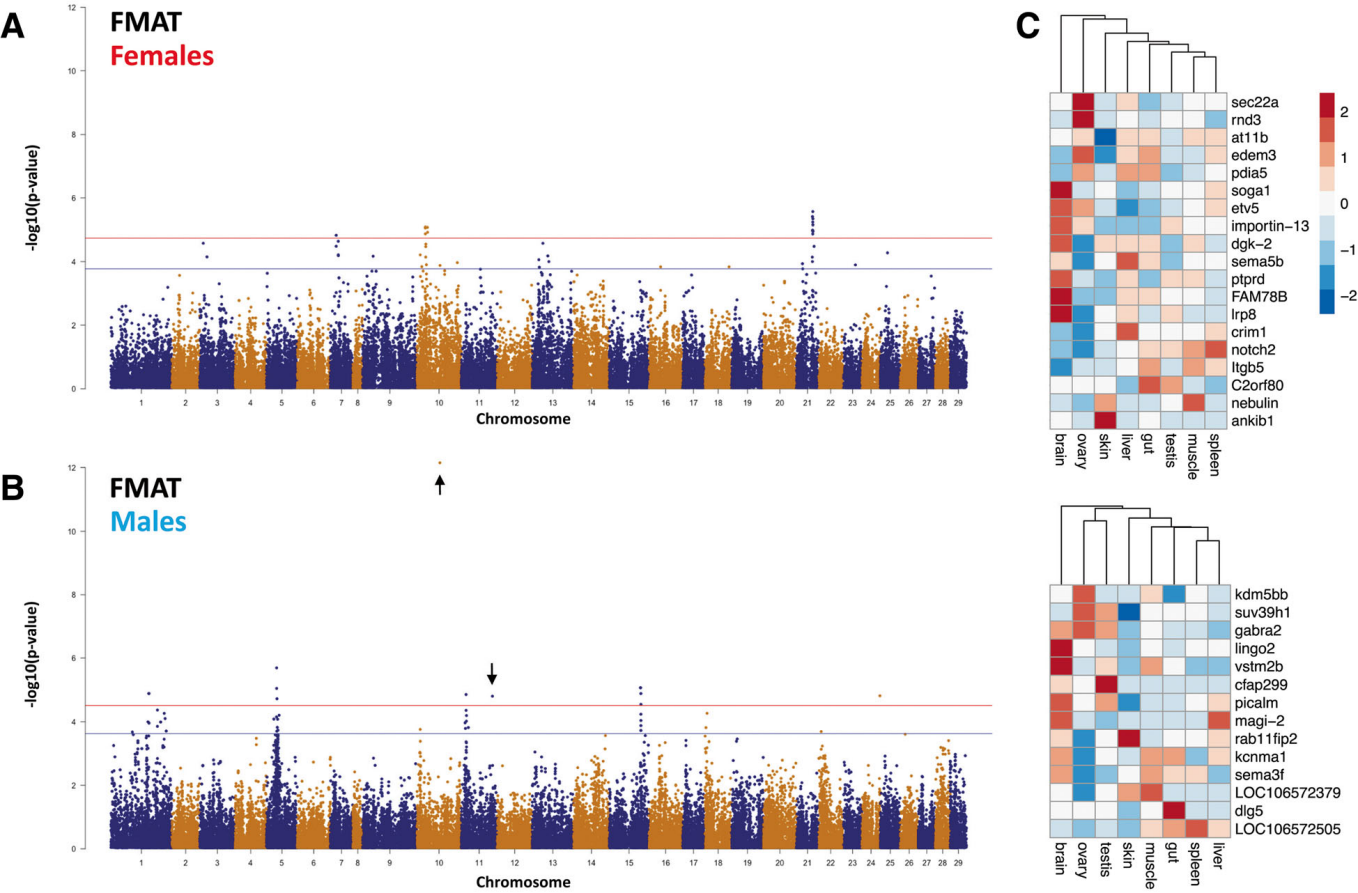
GWAS for freshwater maturation traits conducted separately within females (**a**) and males (**b**). As for Fig. 2, SNP associations are represented as–Log10(*p*-values) and positional genes obtained from GWAS were used to examine the relationship between eight tissues based on gene expression (**c**)

The observation that maturation exhibited sexually dimorphic behaviour (Fig. 1) prompted GWAS using males and females separately. This sought to determine if the association signals obtained using all animals originated predominantly from males, females or the combined contribution of both sexes. Analysis of the freshwater trait revealed 19 and 24 significant SNP in male and female fish respectively (Fig. 3). Male maturation in freshwater was associated with regions on Ssa5, 10, 11 and 15 while SNP were identified in females within regions on Ssa10 (22–27 Mb) and Ssa21 (42–43 Mb) (Additional files 7 and 8). Comparison between the results revealed none of the SNP or regions were shared, suggesting male and female maturation in freshwater are not obviously controlled by the same major genes. Interestingly, the top two loci identified using all animals (*AX-87354755* and *AX-96411005*) were reconstituted in analysis using male fish but absent in the female specific analysis. For example, the proportion of genetic variation explained by the chromosome 10 SNP (*AX-87354755*) was 19.5% in males and non-significant in females despite its allele frequency being similar in both sexes (Additional files 9, 10, 11).

**Fig. 4:**
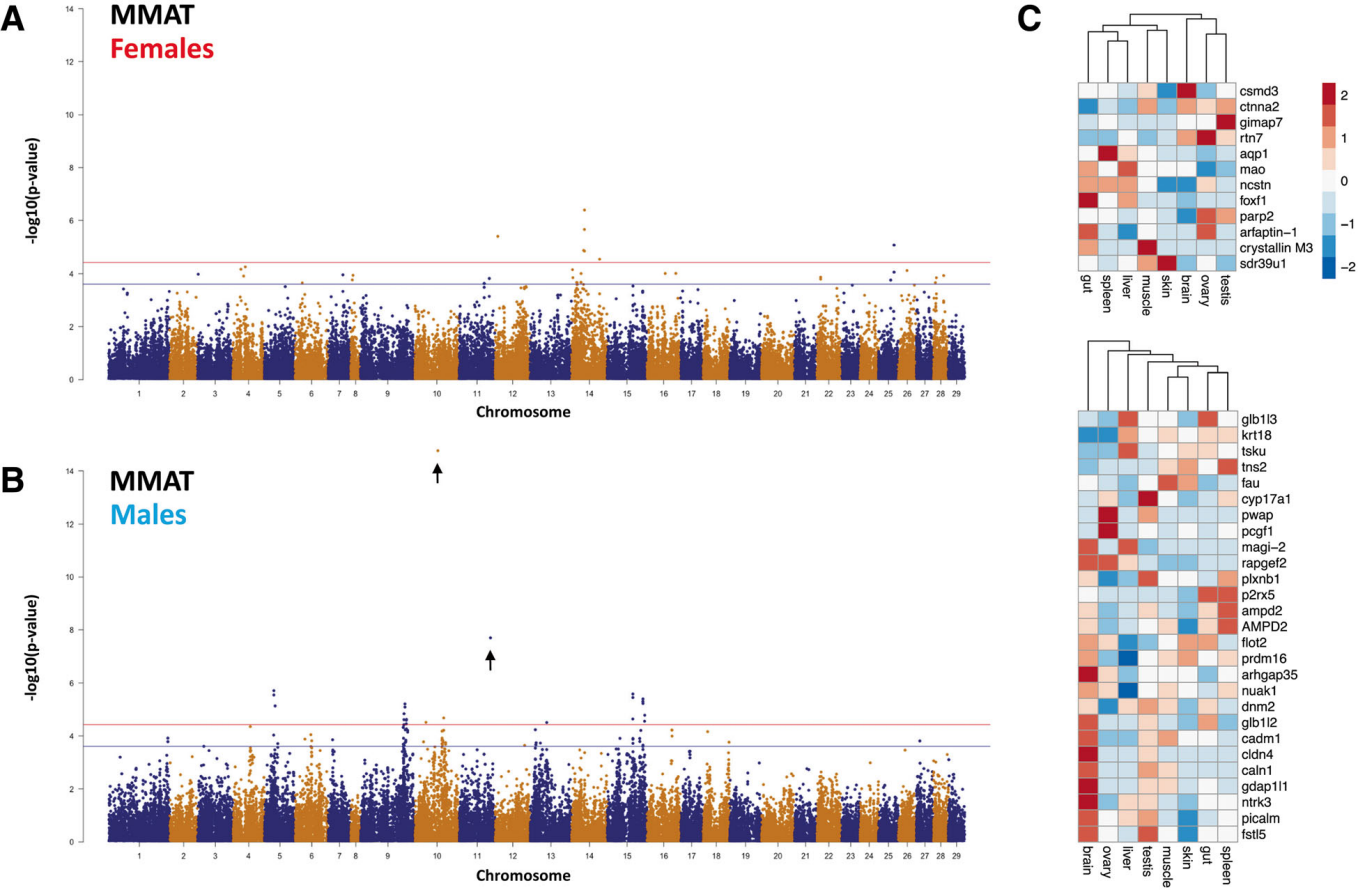
GWAS for marine maturation traits conducted separately within females (**a**) and males (**b**). SNP associations are represented as –Log10(*p*-values) and positional genes obtained from GWAS were used to examine the relationship between eight tissues based on gene expression (**c**)

Sex specific analysis for marine maturation returned broadly similar results (Fig. 4). The two most significantly associated SNP on Ssa10 and 11 within the male population (*AX-87354755* and *AX-96411005*) were again not significant using females alone (Additional files 9, 10, 11). Beyond these two SNP, the majority of 29 significant loci identified in males for MMAT (17 or 58%) overlap the set obtained using both sexes (Additional file 12). This contrasts the observation in females, where only 2 of the 12 significant loci were found in GWAS using all fish (Additional file 13). Taken together, the analysis suggests both maturation traits exhibit a sex specific genetic architecture whereby males more strongly contribute to the GWAS results obtained using both sexes.

### Gene expression characterisation of positional candidates

Sexual maturation is likely to involve genes with non-random tissue expression profiles. Reproductive maturation takes place in the testis and ovary, and is likely mediated by genes with preferential expression in other tissues including the brain and the pituitary [39–41]. This prompted analysis to determine if the genes identified as positional candidates in GWAS using all animals exhibit specialised gene expression profiles. Published Atlantic salmon RNA-Seq data from eight tissues were mapped onto Atlantic salmon genome ISCGA_v2 [23] (Additional file 2), before normalised expression values for 45,531 genes were used to cluster the tissue collection. This revealed two clusters, with the ovary, brain and testis grouping separately to the other five tissues (Fig. 1c). To assess if the positional biological candidates exhibit a different expression profile, the analysis was repeated using the top 10 genes arising from GWAS. For both traits, the expression profiles of the positional candidates returned tissue relationships that differed substantially from that built using all genes. Brain clustered separately to the other seven tissues using genes implicated in marine maturation (Fig. 3d). For FMAT, the expression profile grouped the two reproductive organs (testis and ovary) together and separate from other tissues.

### Analysis of vgll3 and akap11 SNP

The final analysis was prompted by two earlier genetic studies that identified a < 1 Mb region on Ssa25 that controls a large proportion of the variation in the age that wild European Atlantic salmon return mature to spawn in freshwater [11, 42]. Two genes *vgll3* and A-kinase anchoring protein 11 (*akap11*) containing three non-synonymous mutations (*vgll3 Met54Thr*, *vgll3 Asn323Lys* and *akap11 Val214Met*) were identified as putatively functional. Given sea age is associated with the timing of sexual maturity, we sought to determine if variation at either gene is associated with maturation in the Tasmanian breeding population. To commence, whole genome sequence from the same 19 animals used for imputation were used to estimate the allele frequency at each of the three putatively causal variants (Table 3). The most strongly associated *vgll3* variant (*Asn323Lys*) [9] was monomorphic within the SALTAS genomes, with every animal homozygous for the *323Lys* variant associated with delayed maturation and 3 sea winter animals. Both alleles were detected at the second *vgll3* mutation (Met54Thr), with the frequency of the 3 sea winter allele (54Thr) much higher in the SALTAS genomes (0.78) than the frequency for the early maturing allele (0.23). In preparation for fine mapping the *vgll3* region, we incorporated five SNP into the design of the Custom SNP50 chip spanning a 62 Kb interval (Mb position 28.659–28.721). Inspection of the resulting association signals revealed no evidence for variation at, or in the chromosomal regions surrounding *vgll3*, influencing freshwater or marine maturation (Additional file 14). Interestingly, SNP within and immediately flanking the gene displayed complete monomorphism (bp positions 28,703,619, 28,666,898, 28,707,912, 28,720,779 and 28,658,151). This suggested the presence of a selective sweep at the gene, prompting analysis of SNP polymorphism across the chromosome. No evidence of reduced variability or increased homozygosity was observed, indicating the locus has not experienced a hard selection sweep (Additional file 14). The alternative explanation for monomorphism is the *vgll3* associated variants are fixed in North American derived populations due to their divergence from the European stocks in which the polymorphisms were discovered.

**Table 3:** Genotype and allele frequencies are given for three SNP, previously associated with either early (E) or late (L) maturing wild Atlantic salmon [9]

SNP	Genotype Freq			Allele Freq	
	EE	EL	LL	E	L
*vgll3* Met54Thr					
	Met/Met	Met/Thr	Thr/Thr	Met	Thr
TAS	0.10	0.25	0.65	0.23	0.78
NA Wild	0.02	0.22	0.76	0.13	0.87
*vgll3*Asn323Lys					
	Asn/Asn	Asn/Lys	Lys/Lys	Asn	Lys
TAS	0.00	0.00	1.00	0.00	1.00
NA Wild	0.02	0.22	0.77	0.23	0.88
*akap11* Val214Met					
	Val/Val	Val/Met	Met/Met	Val	Met
TAS	1.00	0.00	0.00	1.00	0.00
NA Wild	1.00	0.00	0.00	1.00	0.00

Data is given for 19 fish derived from whole genome sequence [16]. Data from North American (NA) wild fish (n = 1464) is taken from [43]

## Discussion

The high proportion of males entering early maturation, measured here as 55% for marine fish at 22 months, represents a major challenge for the Atlantic salmon farming industries. A key objective of this study was therefore to characterise the distribution of genes underlying variation in maturation, in advance of optimising approaches for selective breeding. GWAS is the best approach to explore the genetic architecture of a given trait and the outcome is difficult to predict beforehand. For example, some disease resistance and life history traits which may be expected to be highly polygenic have proven to be controlled by a small number of major genes in salmon [11, 12, 41], while growth rate appears to be highly polygenic [7, 13]. This study demonstrated both freshwater and marine maturation, as measured in Tasmanian farmed Atlantic salmon, are controlled by a sizable number of loci with none generating exceptionally strong association peaks. We failed to observe SNP explaining a sufficiently large proportion of genetic variation given the low heritabiliy of the traits to warrant the development of dedicated DNA diagnostics for marker assisted selection, or which present compelling targets for gene editing in a research setting to deepen our understanding of sexual maturation. The conclusion relating to the major objective is therefore that genomic prediction is the most efficient method to achieve genetic gain for either trait.

Despite the absence of a single major association peak, the modest number of SNP associations detected are likely to be enriched for true biological drivers of maturation given the sizable number of loci and animals used in the experiment. The low heritability of each trait, coupled with only moderate correlation between them, suggests it was not surprising that few genomic regions were associated independently to both MMAT and FMAT. There were, however, two notable exceptions for loci on Ssa10 and Ssa11 that implicate the genes *picalm* and *magi2* in maturation (Tables 1 and 2). It is worthwhile noting that for both SNP, neighbouring loci failed to exhibit significant association signal to form broad association peaks. This raises the possibility the genomic location of the two peak SNP may not be correct, and suggests caution is required with regard the involvement of the *picalm* and *magi2* genes. Neither gene has been directly implicated in fish maturation previously, however both do represent plausible position candidate genes [34, 35]. Interestingly, both display sex specific behaviour in GWAS whereby the strength of association, effect size and the proportion of genetic variance explained is restricted to males and absent from females. This phenomenon has been observed previously in controlling age at maturity in wild populations of Atlantic salmon, mediated by sex specific dominance patterns at the *vgll3* locus on chromosome 25 [2, 11]. The identification here of sex specific effects at two additional independent loci, within a genetically distinct population and explaining up to 20% of the genetic variance, supports the possibility that sex specific genetic architecture is an important component controlling Atlantic salmon maturation.

A number of earlier studies have sought to identify genes and chromosomal regions controlling salmonid maturation [7, 11, 12]. A key consideration in comparing earlier results to our findings concerns the equivalence of the maturation traits under investigation. A number of critical differences exist including i) the age at which sexual maturation was measured here compared with earlier work; ii) genetic history spanning either European and North American derived populations and iii) variation in environmental factors such as feed availability and photoperiod experienced by farmed versus wild populations. It is worthwhile considering that in this study, two maturation traits measured in one population intentionally managed to remove environmental variation until animals were around a year old still shared a low proportion of significant GWAS hits and displayed only moderate correlation of family means. It is therefore perhaps unrealistic to anticipate the identification of shared genes between this and earlier studies. Despite the differences, we proceeded to characterise the *vgll3* region due to the large effect size it imparts on maturation in both wild European [11] and North American fish [43]. Earlier work prioritised two non-synonymous *vgll3* mutations as most likely to be the functional variant. One is polymorphic and segregating in the Tasmanian population (Met54Thr), the second appears fixed for the allele associated with high maturation age (Asn323Lys), and GWAS clearly showed a lack of association in the region containing *vgll3*. This suggests Met54Thr is not the functional mutation, because if it exerted a phenotypic consequence it should generate an association signal which wasn’t present. Discounting Met54Thr as causal doesn’t mean Asn323Lys must therefore be responsible for controlling the phenotypic variation, however it adds to the weight of evidence in its favour. A more practical implication is there appears to be no benefit in using *vgll3* DNA diagnostics to promote delayed maturation in the Tasmanian breeding program.

## Conclusions

GWAS revealed a highly polygenetic nature for both maturation traits, with few common SNP suggesting they are likely controlled by largely distinct genetic mechanisms. Only two variants were significantly associated with both traits, and each display sex specific effects restricted to male fish. Neither GWAS suggest *vgll3* plays a major role as measured in the Tasmanian population. These results increase our understanding of the genetic basis of maturation and direct future strategies to delay maturation in this important aquaculture species.

## Additional files

Additional file 1: Q-Q plots compared between GWAS methods. The expected distribution of *p*-values is compared to the observed values derived from linear regression (A) and the MLMA-LOCO approach for MMAT (B and C). (TIF 120 kb)

Additional file 2: RNA-Seq data used for analysis of tissue specific expression. (DOCX 13 kb)

Additional file 3: The SALTAS breeding program design. Animals are tagged and sampled for DNA testing at around 10 months of age, before the majority of animals are smolted for transfer to sea cages. The two maturation traits were collected 22 months after spawning. Freshwater progeny are maintained as candidates for the subsequent cycles of the breeding program. (TIF 145 kb)

Additional file 4: All significant SNP identified for freshwater maturation, using both male and female fish. The table shows physically co-located genes and their distance from the associated loci (in bp). Gene identifiers, symbol and names are provided. (XLSX 10 kb)

Additional file 5: All significant SNP identified for marine maturation, using both male and female fish. The table shows physically co-located genes and their distance from the associated loci (in bp). Gene identifiers, symbol and names are provided. (XLSX 13 kb)

Additional file 6: Imputed chromosome Ssa11 SNP surrounding the *picalm* gene and their association to maturation. The table contains associations to both FMAT and MMAT. (DOCX 13 kb)

Additional file 7: All significant SNP identified for freshwater maturation, using only male fish. The table shows physically co-located genes and their distance from the associated loci (in bp). Gene identifiers, symbol and names are provided. (XLSX 10 kb)

Additional file 8: All significant SNP identified for freshwater maturation, using only female fish. The table shows physically co-located genes and their distance from the associated loci (in bp). Gene identifiers, symbol and names are provided. (XLSX 10 kb)

Additional file 9: Sex specific behaviour at two SNP for both FMAT and MMAT. The table shows the strength of association, effect size and proportion of genetic variance explained in analysis using all animals, males or females alone. (DOCX 18 kb)

Additional file 10: Genotype classes by maturation status for Ssa10 SNP AX-87354755. The distribution of genotype classes are shown separately within males and females in both matured and non-matured animals. (TIF 159 kb)

Additional file 11: Genotype classes by maturation status for Ssa11 SNP AX-96411005. The distribution of genotype classes are shown separately within males and females in both matured and non-matured animals. (TIF 162 kb)

Additional file 12: All significant SNP identified for marine maturation, using only male fish. The table shows physically co-located genes and their distance from the associated loci (in bp). Gene identifiers, symbol and names are provided. (XLSX 11 kb)

Additional file 13: All significant SNP identified for marine maturation, using only female fish. The table shows physically co-located genes and their distance from the associated loci (in bp). Gene identifiers, symbol and names are provided. (XLSX 9 kb)

Additional file 14: SNP association for maturation traits on chromosome 25. GWAS for FMAT (A) and MMAT (B) are shown spanning the region containing *VGLL3* (vertical lines). No association peak was evident for either trait. Minor allele frequency (MAF) for SNP was plotted to search for evidence of a selection sweep for FMAT (C) and MMAT (D). No evidence was seen for decreased allele frequency in the region surrounding the gene. Together, this suggests the gene has no effect on maturation as measured in the SALTAS population. (TIF 226 kb)

## Abbreviations

*akap11*: A-kinase anchoring protein 11
BAM: Binary alignment map
CIGENE: Centre of Integrative Genetics
FMAT: Maturation in the freshwater environment
FPKM: Fragments per kilobase of transcript per million mapped reads
GCTA: Genome-wide complex trait analysis
GRM: Genetic relationship matrix
GWAS: Genome wide association studies
magi2: Membrane-associated guanylate kinase, WW and PDZ domain-containing protein 2
MLMA: Mixed linear method analysis
MMAT: Maturation in the marine environment
*picalm*: Phosphatidylinositol-binding clathrin assembly protein-like
SALTAS: Salmon Enterprises of Tasmania
SAM: Sequence alignment map
*sdY*: Sex-determining gene
SNP: Single nucleotide polymorphism
SRA: Sequence Read Archive
TMM: Trimmed mean of M-values
*vgll3*: Vestigial-like family member 3
