# Supplementary figures and images for: Polygenic and sex specific architecture for two maturation traits in farmed Atlantic salmon
Source: BMC Genomics. 2019 Feb 15;20:139. doi: 10.1186/s12864-019-5525-4 (PMC6377724; doi:10.1186/s12864-019-5525-4)

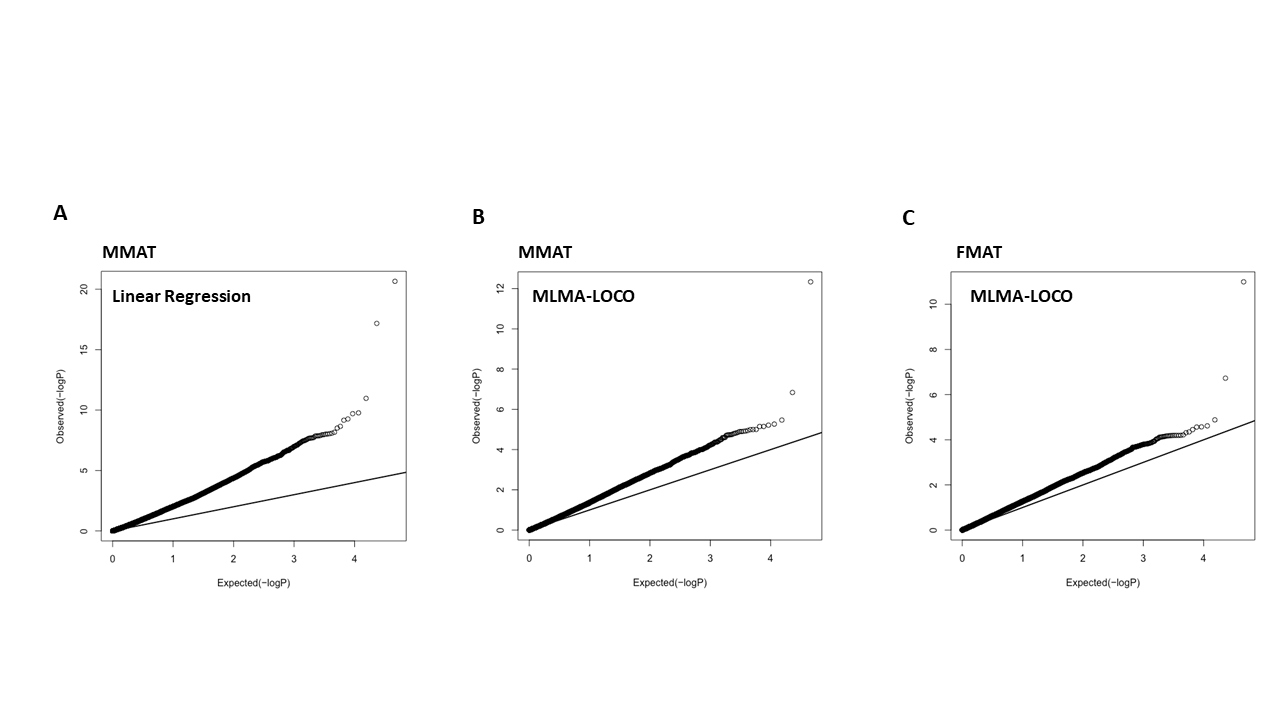

Supplement: Supplementary file 1 — Q-Q plots compared between GWAS methods. The expected distribution of p-values is compared to the observed values derived from linear regression (A) and the MLMA-LOCO approach for MMAT (B and C). (TIF 120 kb) [file 12864_2019_5525_MOESM1_ESM.tif]

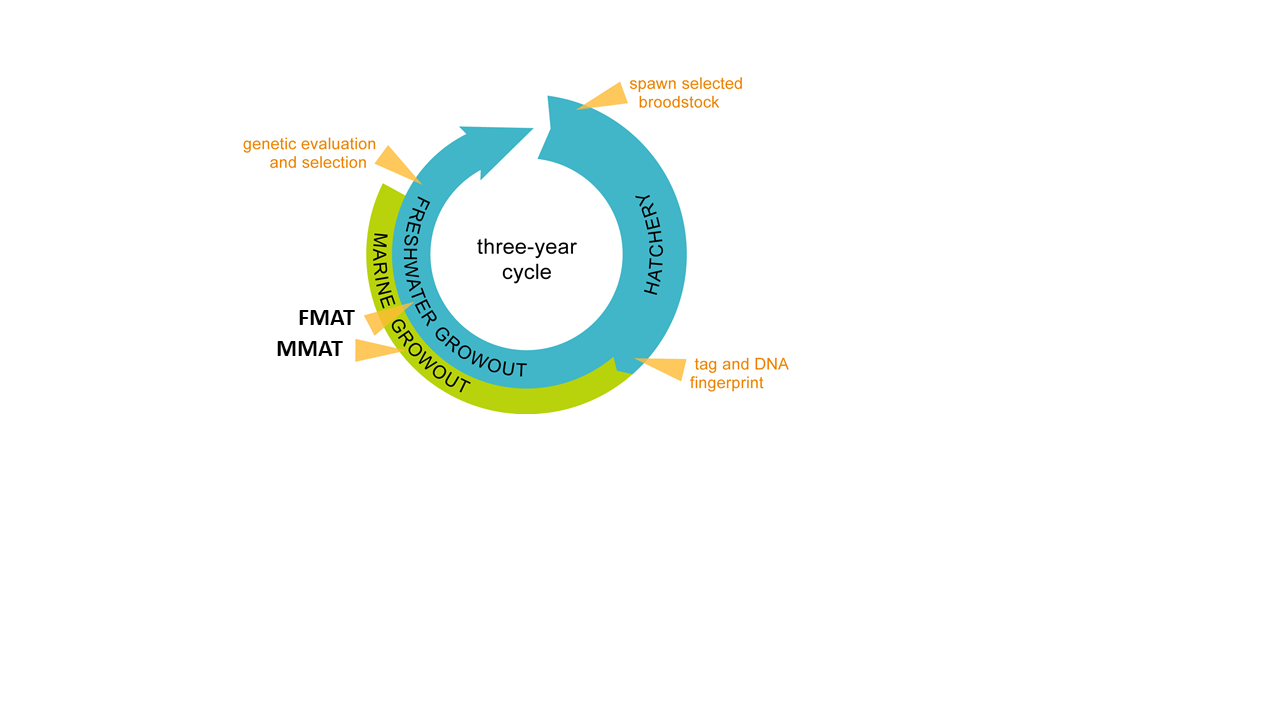

Supplement: Supplementary file 3 — The SALTAS breeding program design. Animals are tagged and sampled for DNA testing at around 10 months of age, before the majority of animals are smolted for transfer to sea cages. The two maturation traits were collected 22 months after spawning. Freshwater progeny are maintained as candidates for the subsequent cycles of the breeding program. (TIF 145 kb) [file 12864_2019_5525_MOESM3_ESM.tif]

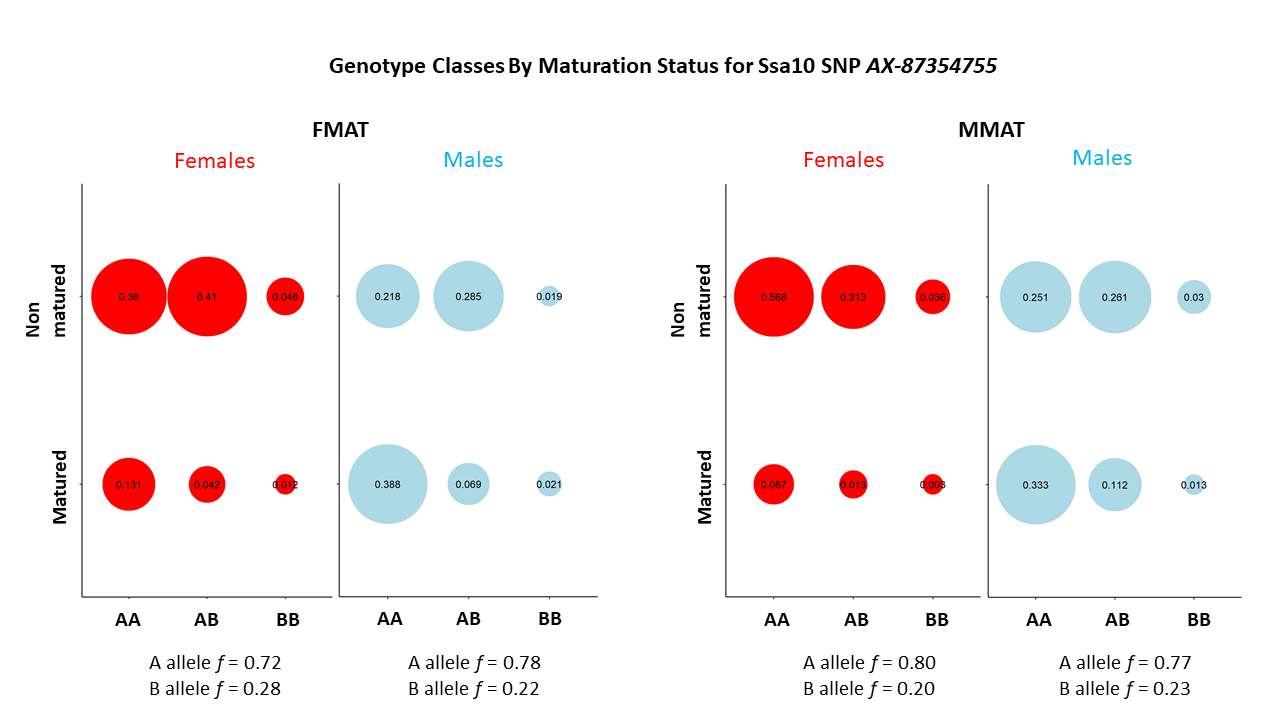

Supplement: Supplementary file 10 — Genotype classes by maturation status for Ssa10 SNP AX-87354755. The distribution of genotype classes are shown separately within males and females in both matured and non-matured animals. (TIF 159 kb) [file 12864_2019_5525_MOESM10_ESM.tif]

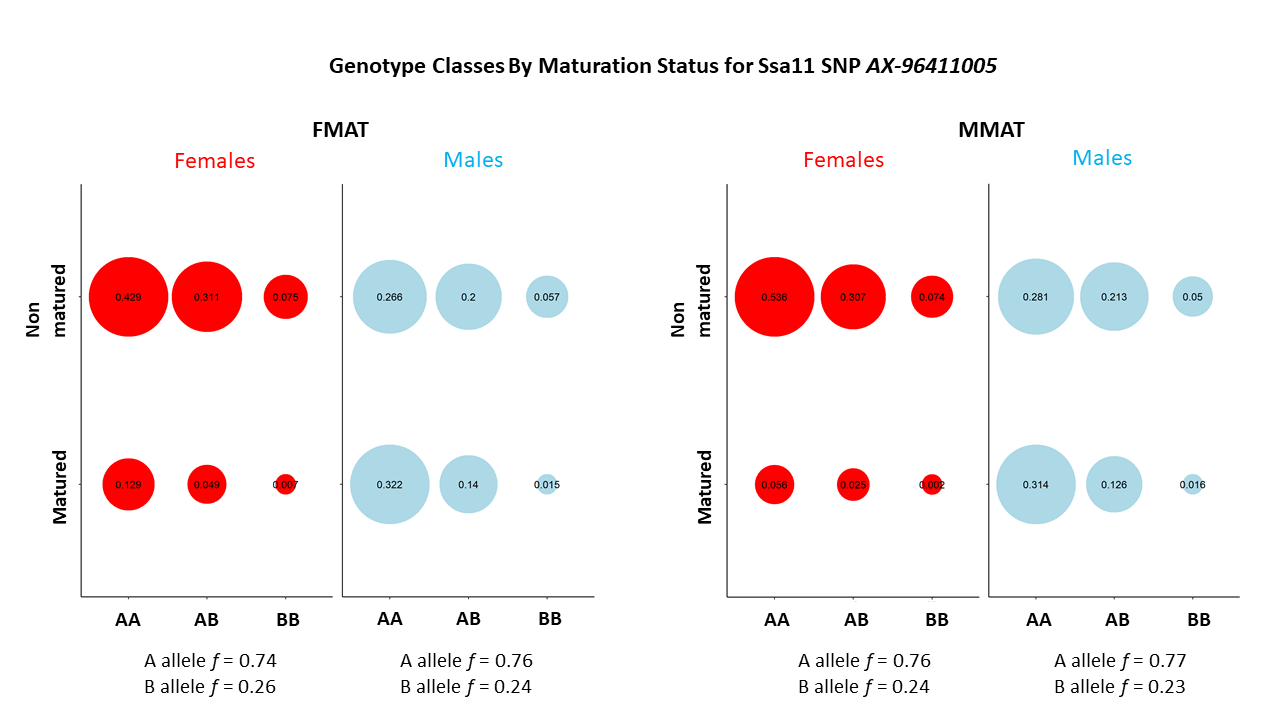

Supplement: Supplementary file 11 — Genotype classes by maturation status for Ssa11 SNP AX-96411005. The distribution of genotype classes are shown separately within males and females in both matured and non-matured animals. (TIF 162 kb) [file 12864_2019_5525_MOESM11_ESM.tif]

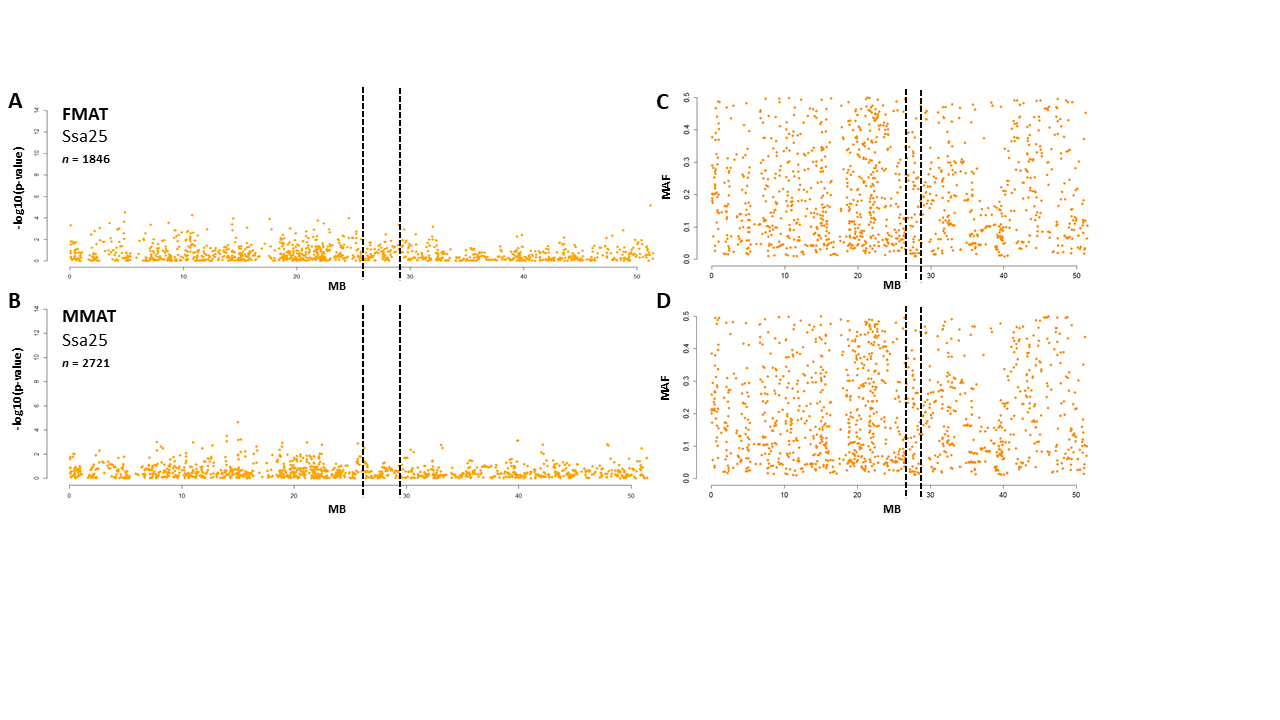

Supplement: Supplementary file 14 — SNP association for maturation traits on chromosome 25. GWAS for FMAT (A) and MMAT (B) are shown spanning the region containing VGLL3 (vertical lines). No association peak was evident for either trait. Minor allele frequency (MAF) for SNP was plotted to search for evidence of a selection sweep for FMAT (C) and MMAT (D). No evidence was seen for decreased allele frequency in the region surrounding the gene. Together, this suggests the gene has no effect on maturation as measured in the SALTAS population. (TIF 226 kb) [file 12864_2019_5525_MOESM14_ESM.tif]
