# Supplementary material for: Polygenic and sex specific architecture for two maturation traits in farmed Atlantic salmon
Source: BMC Genomics. 2019 Feb 15;20:139. doi: 10.1186/s12864-019-5525-4 (PMC6377724; doi:10.1186/s12864-019-5525-4)
Supplement: Supplementary file 2 — RNA-Seq data used for analysis of tissue specific expression. (DOCX 13 kb) [file 12864_2019_5525_MOESM2_ESM.docx]

**Additional file 2**. RNA-Seq data used for specific expression of eight salmon tissues.

| **Tissue Type** | **Brain** | **Gut** | **Liver** | **Muscle** | **Skin** | **Spleen** | **Ovary** | **Testis** |
| --- | --- | --- | --- | --- | --- | --- | --- | --- |
| **Sample SRR ID** | 1422856 | 1422859 | 1422865 | 1422866 | 1422869 | 1422870 | 1422871 | 1422872 |
| **Raw PE reads (M)** | 29.46 | 29.90 | 29.39 | 30.71 | 135.48 | 30.10 | 42.02 | 92.65 |
| **Mapped left reads (M)** | 24.70 | 26.31 | 25.51 | 26.49 | 70.21 | 26.93 | 37.27 | 83.97 |
| **Mapped right reads (M)** | 24.53 | 26.17 | 25.33 | 26.35 | 67.62 | 26.75 | 36.78 | 83.11 |
| **Read mapping rate** | 83.50% | 87.80% | 86.50% | 86.00% | 50.90% | 89.20% | 88.10% | 90.20% |

Raw numbers of paired-end (PE) Illumina reads are shown in millions (M). Following mapping the raw reads against Atlantic salmon reference genome (ICSASG_v2), the percentage of mapped reads is given for each tissue. The data was originally published as Lien et al 2016).
