# Supplementary material for: Polygenic and sex specific architecture for two maturation traits in farmed Atlantic salmon
Source: BMC Genomics. 2019 Feb 15;20:139. doi: 10.1186/s12864-019-5525-4 (PMC6377724; doi:10.1186/s12864-019-5525-4)
Supplement: Supplementary file 6 — Imputed chromosome Ssa11 SNP surrounding the picalm gene and their association to maturation. The table contains associations to both FMAT and MMAT. (DOCX 13 kb) [file 12864_2019_5525_MOESM6_ESM.docx]

**Additional file 6**: Imputed chromosome Ssa11 SNP surrounding the *picalm* gene and their association to maturation.

|  |  |  |  |  |  |
| --- | --- | --- | --- | --- | --- |
| CHR | SNP | FMAT_UN | FMAT_B | MMAT_UN | MMAT_B |
| 11 | 81517947 | 14.52 | 11.86 | 10.57 | 7.91 |
| 11 | 81521399 * | 14.52 | 11.86 | 10.57 | 7.91 |
| 11 | 81522071 | 14.52 | 11.86 | 10.57 | 7.91 |
| 11 | 81524748 | 14.52 | 11.86 | 10.57 | 7.91 |
| 11 | 81525422 | 14.52 | 11.86 | 10.57 | 7.91 |
| 11 | 81525602 | 14.52 | 11.86 | 10.57 | 7.91 |
| 11 | 81525673 | 14.52 | 11.86 | 10.57 | 7.91 |
| 11 | 81525867 | 14.52 | 11.86 | 10.57 | 7.91 |
| 11 | 81525869 | 14.52 | 11.86 | 10.57 | 7.91 |
|  |  |  |  |  |  |

The 9 SNP listed have the most extreme association of 458 imputed variants tested spanning a 3 Mb region (Mb 80 – 83). The SNP have the same uncorrected –Log10(p-value) or bonferroni corrected –Log10(p-value) values (_B) for either freshwater (FMAT) or marine maturation (MMAT) due to complete linkage disequilibrium (*r*^2^ = 1). SNP identifiers give the base pair location. The asterisk identifies the SNP (*AX-87621437*) present on the 50K array which returned the genome wide peak association to MMAT and second highest strongest for FMAT.
