# Supplementary material for: Polygenic and sex specific architecture for two maturation traits in farmed Atlantic salmon
Source: BMC Genomics. 2019 Feb 15;20:139. doi: 10.1186/s12864-019-5525-4 (PMC6377724; doi:10.1186/s12864-019-5525-4)
Supplement: Supplementary file 9 — Sex specific behaviour at two SNP for both FMAT and MMAT. The table shows the strength of association, effect size and proportion of genetic variance explained in analysis using all animals, males or females alone. (DOCX 18 kb) [file 12864_2019_5525_MOESM9_ESM.docx]

**Additional file 9**: Sex specific behaviour at two SNP for both FMAT and MMAT.

| **Chr** | **SNP** | **Trait** | **Pop** | **Freq** | **Effect Size** | **se** | **-Log10**  **(p_value)** | **% V_G_** |
| --- | --- | --- | --- | --- | --- | --- | --- | --- |
|  |  |  |  |  |  |  |  |  |
| 10 | *AX-87354755* | FMAT | ALL | 0.261 | -0.13 | 0.02 | 11.00 | 17.69 |
|  |  |  | MALE | 0.217 | -0.27 | 0.04 | 12.15 | 19.44 |
|  |  |  | FEMALE | 0.284 | NS | NS | NS | NS |
|  |  |  |  |  |  |  |  |  |
|  |  | MMAT | ALL | 0.214 | -0.11 | 0.01 | 12.34 | 2.52 |
|  |  |  | MALE | 0.229 | -0.22 | 0.03 | 14.77 | 16.43 |
|  |  |  | FEMALE | 0.202 | NS | NS | NS | NS |
|  |  |  |  |  |  |  |  |  |
| 11 | *AX-96411005* | FMAT | ALL | 0.255 | -0.08 | 0.02 | 6.73 | 4.63 |
|  |  |  | MALE | 0.242 | -0.14 | 0.03 | 4.81 | 0.55 |
|  |  |  | FEMALE | 0.262 | NS | NS | NS | NS |
|  |  |  |  |  |  |  |  |  |
|  |  | MMAT | ALL | 0.239 | -0.07 | 0.01 | 6.84 | 2.64 |
|  |  |  | MALE | 0.236 | -0.14 | 0.02 | 7.70 | 2.45 |
|  |  |  | FEMALE | 0.242 | NS | NS | NS | NS |
|  |  |  |  |  |  |  |  |  |

The two top ranked SNP in GWAS exhibited sex specific association (given as the –Log10(p-value). GWAS was performed using three populations (Pop), including all fish or males and females separately. The population specific frequency of the minor allele (Freq) is given, along with the effect size and it’s standard error (se). Effect size is derived from single SNP GWAS using the categoric assignment of mature or non-matured animals (0 or 1) at 22 months of age. The proportion of genetic variance, estimated using a model fitting all significant SNP with both pedigree and SNP derived GRM, is shown as %V_G_. Non significance is indicated as ‘NS’.
